# A New SARS-CoV-2 Dual-Purpose Serology Test: Highly Accurate Infection Tracing and Neutralizing Antibody Response Detection

**DOI:** 10.1128/JCM.02438-20

**Published:** 2021-03-19

**Authors:** Sean C. Taylor, Beth Hurst, Carmen L. Charlton, Ashley Bailey, Jamil N. Kanji, Mary K. McCarthy, Thomas E. Morrison, Leah Huey, Kyle Annen, Melkon G. DomBourian, Vijaya Knight

**Affiliations:** a GenScript USA Inc., Piscataway, New Jersey, USA; b Cayman Chemical, Ann Arbor, Michigan, USA; c Public Health Laboratory, Alberta Precision Laboratories (ProvLab), University of Alberta Hospital, Edmonton, AB, Canada; d Department of Laboratory Medicine and Pathology, University of Alberta, Edmonton, AB, Canada; e Li Ka Shing Institute of Virology, University of Alberta, Edmonton, AB, Canada; f Division of Infectious Diseases, Department of Medicine, University of Alberta, Edmonton, AB, Canada; g Department of Immunology and Microbiology, University of Colorado School of Medicine, Aurora, Colorado, USA; h Department of Pediatrics, University of Colorado School of Medicine, Aurora, Colorado, USA; i Department of Pediatrics, Children’s Hospital Colorado Anschutz Medical Campus, Aurora, Colorado, USA; j Department of Pathology and Laboratory Medicine, University of Colorado School of Medicine, Aurora, Colorado, USA; k Department of Pathology and Laboratory Medicine, Children’s Hospital Colorado Anschutz Medical Campus, Aurora, Colorado, USA; St. Jude Children’s Research Hospital

**Keywords:** ELISA, SARS-CoV-2, neutralizing antibodies, serology, vaccines

## Abstract

Many severe acute respiratory syndrome coronavirus 2 (SARS-CoV-2) serology tests have proven to be less accurate than expected and do not assess antibody function as neutralizing, correlating with protection from reinfection. A new assay technology measuring the interaction of the purified SARS-CoV-2 spike protein receptor binding domain (RBD) with the extracellular domain of the human angiotensin-converting enzyme 2 (hACE2) receptor detects these important antibodies. The cPass surrogate virus neutralization test (sVNT), compared directly with eight SARS-CoV-2 IgG serology and two live-cell neutralization tests, gives similar or improved accuracy for qualitative delineation between positive and negative individuals in a fast, scalable, and high-throughput assay. The combined data support the cPass sVNT as a tool for highly accurate SARS-CoV-2 immunity surveillance of infected/recovered and/or vaccinated individuals as well as drug and convalescent-phase donor screening. The data also preview a novel application for the cPass sVNT in calibrating the stringency of live-cell neutralization tests and its use in longitudinal testing of recovered and/or vaccinated patients.

## Abstract (precis)

Many severe acute respiratory syndrome coronavirus 2 (SARS-CoV-2) serology tests have proven to be less accurate than expected and do not assess antibody function as neutralizing, correlating with protection from reinfection. A new assay technology measuring the interaction of the purified SARS-CoV-2 spike protein receptor binding domain (RBD) with the extracellular domain of the human angiotensin-converting enzyme 2 (hACE2) receptor detects these important antibodies.

## INTRODUCTION

Molecular and serological tests for severe acute respiratory syndrome coronavirus 2 (SARS-CoV-2) are a critical component of disease control strategies globally (1, 2). Consequently, the demand for test kits is high, and the market has responded with a growing number of commercially available tests but without clear global guidelines to ensure their efficacy and accuracy (3). Unfortunately, the data generated from these tests vary widely in terms of sensitivity, specificity, and accuracy, leading to concerns regarding the actual number of disease carriers who can unknowingly spread the virus throughout the population (4–6).

The majority of serology tests on the market primarily detect the natural IgM and IgG antibodies that are generated in response to SARS-CoV-2 infection (5). These assays are typically enzyme-linked immunosorbent assay (ELISA) based, with the plate surface coated with either full-length or truncated purified spike or nucleocapsid protein, with detection via anti-IgG or anti-IgM conjugated to horseradish peroxidase (HRP) or other fluorophores (7). In fact, many of these kits use plates coated with the receptor binding domain (RBD) of the spike protein due to its high immunogenicity (8, 9). Since the coating process involves the passive adsorption of proteins via hydrophobic interactions, conformational changes of the coated protein molecules may occur, resulting in newly exposed or altered epitopes that may not be present in the native state (10–14). This can lead to nonspecific binding of immunoglobulins to the coated surface and reduced specificity (11, 15).

**FIG 1:**
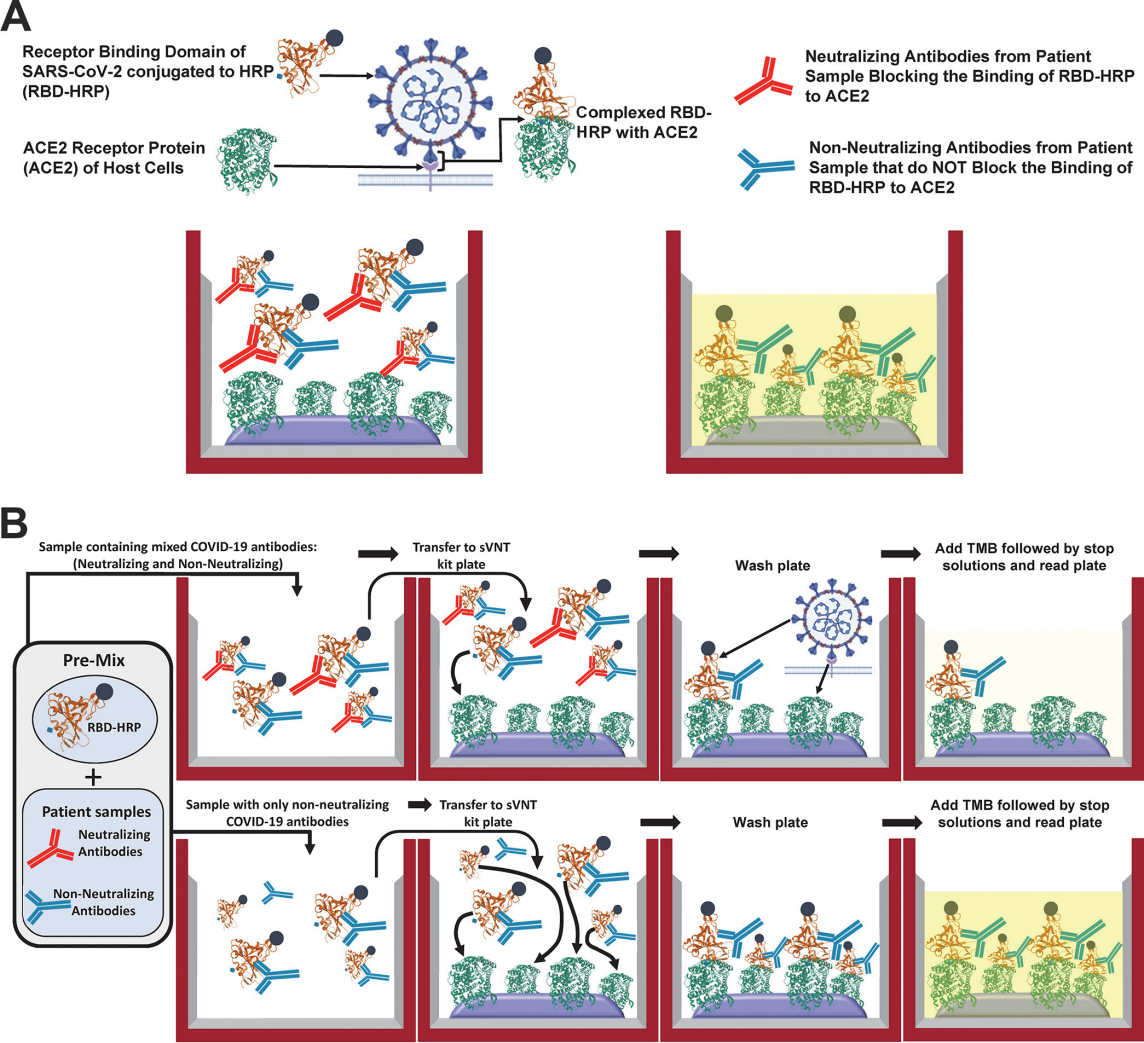
cPass sVNT design and description. (A) sVNT design. The test consists of a purified RBD-HRP conjugate (brown) in solution and ELISA plates coated with the hACE2 receptor (green), which form a strong complex. When mixed with a sample containing proteins, small molecules, or antibodies that block the interaction between the RBD and the hACE2 receptor, a low OD_450_ will be measured after incubation with TMB and stop solution. (B) Performing the sVNT. Sample dilutions are initially mixed with the RBD-HRP solution, with incubation for 30 min at 37°C to permit the binding of components to the RBD. If the sample does not contain constituents that bind and block the RBD-hACE2 interaction (bottom four wells), the RBD-HRP will bind to the hACE2-coated wells, giving a yellow color after incubation with TMB for 15 min at 37°C followed by stop solution. If the sample contains blocking constituents, they will bind to the RBD during the initial 30 min and inhibit the interaction with hACE (top four wells), giving a light-yellow color after the addition of stop solution.

A novel serology assay termed the cPass surrogate virus neutralization test (sVNT) directly addresses the potential problems associated with the preexisting technologies while providing additional functional data (16). Utilizing 96-well plates coated with the purified extracellular domain of the human angiotensin-converting enzyme 2 (hACE2) receptor and a purified, solubilized, recombinant RBD conjugated to HRP (RBD-HRP), the assay capitalizes on the strong interaction between the hACE2 receptor and the RBD coupled with the high immunogenicity of the RBD (8, 17). This permits the direct assessment of the inhibitory capacity of immunoglobulins, antibody-based drugs, and compounds that block (or neutralize) this binding event (Fig. 1) (8, 9, 17, 18).

There are several advantages to this assay format over traditional serology tests. It measures the interaction between the hACE2 receptor and the RBD to elucidate the function of antibodies (and other molecules) as neutralizing (16). The test is amenable to the indirect detection of immunoglobulins that abrogate the interaction between the RBD and the hACE2 receptor and is therefore not specific to any isotype (e.g., IgG, IgM, or IgA, etc.) (i.e., isotype agnostic). Antibodies generated in all species infected with SARS-CoV-2 are detectable with this assay (16, 19). For vaccine and drug development organizations, this test offers potential application as a high-throughput, safe, and practical methodology for screening antibodies, proteins, peptides, or small molecules that block the interaction between the RBD and the hACE2 receptor. As opposed to the more traditional virus neutralization tests (20), this assay does not require a biosafety level 3 (BSL3) containment laboratory. Also, the cPass sVNT can be performed in about 1.5 h per 96-well plate, compared with 2 to 4 days for virus and pseudovirus tests.

The cPass sVNT was compared to eight traditional SARS-CoV-2 IgG ELISAs in two separate studies that utilized protein-coated plates and to two cell-based, live-virus neutralization tests using human serum and plasma samples collected from several cohorts of SARS-CoV-2 PCR-confirmed positive, negative, and prepandemic deidentified samples. Finally, an approach to the use of the cPass sVNT for longitudinal studies to assess changes in neutralizing antibody titers in patients who recovered from coronavirus disease 2019 (COVID-19) or vaccinated subjects is described.

## MATERIALS AND METHODS

### Samples.

**FIG 2:**
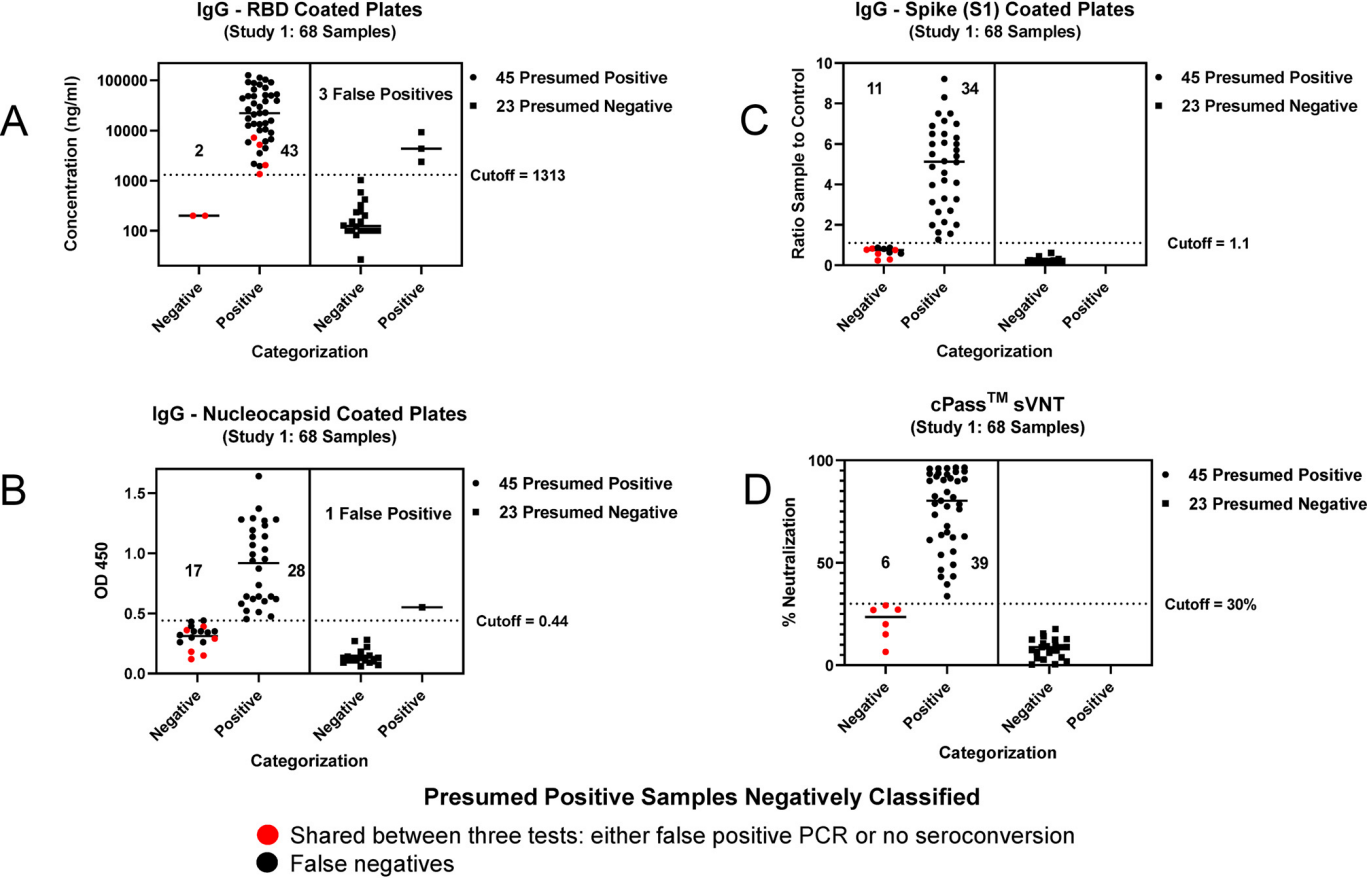
Study 1: direct comparison between nucleocapsid-, RBD-, and spike (S1)-coated IgG ELISA plates with the cPass sVNT. (A) RBD-coated plate; (B) nucleocapsid-coated plate; (C) spike (S1)-coated plate; (D) cPass sVNT. Forty-five PCR-positive samples from patients with blood drawn more than 14 days after PCR testing were categorized by the four tests (round symbols at the left side of each chart). Of the negative delineated samples, six were shared between three tests (red dots). Twenty-three prepandemic presumed negative samples were categorized by the four tests (square symbols at the right side of each chart). Both the nucleocapsid- and RBD-coated IgG ELISA plates gave false positives.

For study 1, plasma and serum samples from The Children’s Hospital Colorado’s COVID-19 convalescent-phase plasma (CCP) donor program registered with the FDA as eligible to collect CCP on 31 March 2020 were collected. Eligible individuals for the CCP donor program were confirmed to be PCR positive for SARS-CoV-2, were symptom free for at least 14 days prior to plasma donation, and met all standard blood donation criteria according to FDA requirements. For each donor, the number of days from the PCR-positive SARS-CoV-2 test to the day of plasma donation and the number of donations were tracked. Positive and prepandemic presumed negative samples were deidentified, tested, and tabulated (Fig. 2 and Table 1).

**TABLE 1:** Combined data from study 1 comparing assay performances of three commercial serology tests and the cPass sVNT^*a*^

Parameter	Value for IgG detection in study 1							
	Nucleocapsid-coated plates		Spike (S1)-coated plates		RBD-coated plates		cPass sVNT	
	Positive (*n* = 45)	Negative (*n* = 23)	Positive (*n* = 45)	Negative (*n* = 23)	Positive (*n* = 45)	Negative (*n* = 23)	Positive (*n* = 45)	Negative(*n* = 23)
No. of positive samples	28	1	34	0	43	3	39	0
No. of negative	17	22	11	23	2	20	6	23
Sensitivity (%)	62.22		75.56		95.56		86.67	
Specificity (%)		95.65		100.00		86.96		100.00
Accuracy (%)	92.31		97.36		87.82		98.67	
PPV (%)	61.39		100.00		44.87		100.00	
NPV (%)	95.80		97.36		99.44		98.54	

a See Fig. 2. Sensitivity, specificity, accuracy, positive predictive values (PPV), and negative predictive values (NPV) are shown. A prevalence of 10% was used for the calculations.

For study 2, a subset of the identical serum samples collected for a previously published article comparing six commercial serology assays were tested and delineated with the cPass sVNT (21). The collection and description of the deidentified patient cohorts for both the positive and prepandemic samples are well described (21). The data for the positive samples from patients between 48 and 80 years of age were grouped, summarized, and tabulated by sampling days after symptom onset along with the prepandemic samples (Table 2).

**TABLE 2:** Combined data from study 2 comparing the assay performances of six commercial serology tests and the cPass sVNT^*a*^

Assay	Analyte	Time after symptom onset																	Negative samples (serum collected before Nov 2019)					PPV (%)	NPV (%)	ACC (%)
		5–9 days				10–19 days				>19 days				All time points					No. of Neg samples	No. of Equ samples	No. of Pos samples	Spec (%)	CI (%)			
		No. of Neg samples	No. of Pos samples	Sens (%)	CI (%)	No. of Neg samples	No. of Pos samples	Sens (%)	CI (%)	No. of Neg samples	No. of Pos samples	Sens (%)	CI (%)	No. of Neg samples	No. of Pos samples	Sens (%)	CI (%)	Total no. of samples								
Abbott	IgG	0	6	100	54–100	3	9	75	43–95	0	9	100	66–100	3	24	89	71–98	27	49	0	1	98	89–100	83	99	97
Affinity	IgG	0	7	100	59–100	2	11	85	55–98	0	8	100	63–100	2	26	93	71–98	28	47	0	0	100	92–100	100	99	99
Bio-Rad	IgG	0	6	100	54–100	3	9	75	43–95	0	9	100	66–100	3	24	89	71–98	27	50	0	0	100	93–100	100	99	99
DiaSorin	IgG	4	2	33	4–78	4	8	67	35–90	0	9	100	66–100	8	19	70	71–98	27	48	1	1	96	86–100	66	97	93
Euroimmun	IgG	3	3	50	12–88	4	8	67	35–90	0	8	100	63–100	7	19	73	71–98	26	50	0	0	100	93–100	100	97	97
Roche	Total Ab	1	6	86	42–100	4	9	69	39–91	0	9	100	66–100	5	24	83	71–98	29	50	0	0	100	93–100	100	98	98
GenScript	RBD-hACE2	1	6	86	42–100	1	12	92	64–100	0	9	100	66–100	2	27	93	71–98	29	50	0	0	100	93–100	100	99	99

a Sensitivity (Sens), specificity (Spec), accuracy (ACC), positive predictive values (PPV), and negative predictive values (NPV) are shown. Pos, positive; Neg, negative; CI, 95% confidence interval; Equ, equivocal result; Ab antibody. A prevalence of 10% was used for the calculations.

The data in Table 3 were derived from PCR-positive and -negative deidentified samples collected and tested in Singapore (Health Sciences Authority, conducted by Diagnostic Development Hub [DxD Hub]), DukeNUS, commercial vendors, and Granger Genetics, with data collated and analyzed by Corgenix Clinical Laboratory.

**TABLE 3:** Combined clinical data for the cPass sVNT^*a*^

GenScript cPass SARS-CoV-2 sVNT parameter	RT-qPCR result	
	Positive (*n* = 186)	Negative (*n* = 480)
No. of positive results	181	3
No. of negative results	5	477
Sensitivity (%)	97.30	
Specificity (%)		99.40
Accuracy (%)	99.20	
PPV (%)	94.50	
NPV (%)	99.70	

a Based on overall clinical data collected by 1 June 2020. Totals of 186 positive and 480 negative samples were verified by PCR and then screened by the sVNT. Sensitivity, specificity, accuracy, and positive and negative predictive values are shown. A prevalence of 10% was used for the calculations.

### SARS-CoV-2 IgG ELISAs.

** (i) Study 1.** The CE-marked Epitope Diagnostics Inc. (EDI) (San Diego, CA) ELISA (catalog number KT-1032) utilizes the SARS-CoV-2 recombinant nucleocapsid antigen, and samples were diluted, tested, and analyzed according to the kit instructions for IgG. The CE-marked and FDA emergency-use authorization (EUA)-approved Euroimmun (Lubeck, Germany) ELISA (catalog number 2606) utilizes the S1 domain, including the receptor binding domain (RBD), of the SARS-CoV-2 spike protein, and samples were diluted, tested, and analyzed according to the kit instructions for IgG. The FDA Policy D, *in vitro* diagnostic (IVD)-status Akston Biosciences (Beverly, MA) ELISA (catalog number 600016) utilizes the recombinant RBD antigen of the SARS-CoV-2 spike protein, with samples diluted, tested, and analyzed according to the kit instructions for IgG.

For the EDI assay, positive, negative, and borderline results were calculated based on the average optical density at 450 nm (OD_450_) value for the negative control assayed in triplicate for the specific assay. The positive cutoff value was calculated using the formula positive cutoff = 1.1 × (NC + 0.18), where NC is the average OD_450_ of triplicate negative-control OD values. For study 1, given the day-to-day fluctuation in OD_450_ values from both the positive cutoff and our own interplate positive-control calibrator, the median positive cutoff OD_450_ for several days of testing (0.44) was used to delineate positive and negative samples.

**(ii) Study 2.** The six commercial IgG ELISAs (Abbott Laboratories, Epitope Diagnostics Inc., Affinity Diagnostics Corp, DRG International Inc. [supplied by Bio-Rad], Euroimmun, and Roche Diagnostics) used for the detection of SARS-CoV-2 IgG antibodies and the associated protocols employed for screening the positive and negative study samples were previously described (21). The positive cutoff defined in the kit instructions for each assay was used to delineate positive and negative samples compared with the cPass sVNT using a 30% cutoff.

### SARS-CoV-2 cPass surrogate virus-neutralizing test.

The GenScript (Piscataway, NJ) cPass sVNT (catalog number L00847) utilizes the recombinant RBD of the SARS-CoV-2 spike protein to detect antibodies that block the RBD from binding to the hACE2 receptor. Plasma or serum samples and the kit-supplied positive and negative controls were diluted 1:10 in kit-specific sample dilution buffer according to the kit insert. The diluted samples and controls were preincubated with RBD-HRP in a “neutralization reaction” mixture for 30 min at 37°C, permitting the interaction and binding of neutralizing antibodies with RBD-HRP (Fig. 1B). Each neutralization reaction mixture was then added to the capture plate precoated with the hACE2 protein whereby the free RBD-HRP as well as RBD-HRP bound to nonneutralizing antibodies strongly interact with hACE2 and were captured on the plate (Fig. 1B). RBD-HRP complexed with neutralizing antibodies (i.e., those blocking the interaction between the RBD and hACE2) remained in the supernatant and were removed in a subsequent wash step. After the wash steps, 3,3′,5,5′-tetramethylbenzidine (TMB) followed by stop solution was added to all wells, permitting the visualization of RBD-HRP bound to the plate based on the OD_450_ intensity. The color intensity is inversely proportional to the amount of neutralizing antibody in standards or samples (Fig. 1B).

Data are interpreted by the percent inhibition of RBD-HRP binding, calculated as follows: percent inhibition = (1 − OD value of sample/OD value of background) × 100%. A 30% cutoff is used to delineate positive and negative samples where this cutoff has been calibrated against the gold-standard plaque reduction neutralization test (PRNT) using high-stringency PRNT_90_ (90% plaque reduction) data analysis. Percent inhibition of ≥30% indicates the presence of SARS-CoV-2 RBD-interacting antibodies blocking the RBD-hACE2 interaction.

### Plaque reduction neutralization test.

The PRNT is considered the gold standard for characterizing neutralizing antibodies to most viruses, including SARS-CoV-2. Serum samples were heat inactivated for 30 min at 56°C. Serial 2-fold dilutions of the inactivated samples were prepared in a 96-well plate (Greiner Bio-One, Monroe, NC). A viral stock (strain hCoV-19/USA/WA1/2020; BEI Resources, Manassas, VA) containing approximately 200 PFU per 0.1 ml was added to each well containing serum dilutions. Following a 1-h incubation period at 37°C in a CO_2_ incubator, 6-well plates (Greiner Bio-One, Monroe, NC) containing recently confluent Vero cells (ATCC, Manassas, VA) were inoculated with the virus-serum mixtures. After a second incubation period of 45 min at 37°C in a CO_2_ incubator, 2 ml of an overlay (2× agarose [melt 1% agarose in water using a microwave oven and cool to 45°C prior to mixing with 2× minimal essential medium {MEM} with 4% fetal bovine {FBS} {Peak Serum, Wellington, CO} and 3 ml of 7.5% sodium bicarbonate per 100 ml of solution]) was added to each well. Finally, the plates were incubated for 24 h at 37°C in a CO_2_ incubator, upon which a second overlay containing neutral red (Millipore Sigma, St. Louis, MO) was dispensed into each well, followed by a 24-h incubation at 37°C in a CO_2_ incubator. The number of plaques was counted 48 to 72 h after the initial inoculation. The highest dilution of serum that inhibits (reduces) plaque formation by 50%, 75%, or 90% (PRNT_50_, PRNT_75_, or PRNT_90_, respectively) was calculated based on the titer of the viral stock and the number of plaques present at each dilution.

### Focus reduction neutralization assay.

For the focus reduction neutralization test (FRNT), Vero E6 cells (ATCC, Manassas, VA) were seeded into 96-well plates. Serum samples were heat inactivated and serially diluted (2-fold, starting at 1:10) in Dulbecco’s modified Eagle’s medium (DMEM) (Thermo Fisher, Pittsburgh, PA, USA) plus 1% FBS in 96-well plates. Approximately 100 focus-forming units (FFU) of SARS-CoV-2 USA-WA1/2020 (deposited by the Centers for Disease Control and Prevention and obtained through BEI Resources, NIAID, NIH) were added to each well, and the serum-virus mixture was incubated for 1 h at 37°C. After incubation, medium was removed from cells, and the serum-virus mixture was added for 1 h at 37°C. After 1 h, samples were removed, and cells were overlaid with 1% methylcellulose (Millipore Sigma, St. Louis, MO) in MEM (Thermo Fisher, Pittsburgh, PA, USA)–2% FBS and incubated for 30 h at 37°C. Cells were fixed with 4% paraformaldehyde (Acros Organics, Pittsburgh, PA, USA) and probed with 1 μg/ml of an anti-SARS-CoV spike monoclonal antibody (CR3022; Absolute Antibody, Boston, MA, USA) in Perm wash (1× phosphate-buffered saline [PBS]–0.1% saponin–0.1% bovine serum albumin [BSA]) for 2 h at room temperature (RT). After washing, cells were incubated with horseradish peroxidase (HRP)-conjugated goat anti-human IgG (Southern Biotech, Birmingham, AL, USA) (1:1,000) for 1.5 h at RT. After washing, SARS-CoV-2-positive foci were visualized with TrueBlue substrate (Thermo Fisher, Pittsburgh, PA, USA) and counted using a CTL Biospot analyzer and Biospot software (Cellular Technology Ltd., Shaker Heights, OH, USA). The FRNT_50_, FRNT_75_, and FRNT_90_ titers were calculated relative to a virus-only control (no serum) set at 100%, using GraphPad Prism 8 (GraphPad, La Jolla, CA, USA) default nonlinear curve fit constrained between 0 and 100%.

## RESULTS

### RBD soluble cPass sVNT versus eight IgG-specific serology tests, including RBD-, nucleocapsid-, or spike (S1)-coated plates, for COVID-19 diagnosis and classification.

For study 1, 68 (45 SARS-CoV-2 PCR presumed positive and 23 prepandemic presumed negative) human serum samples were directly compared across four tests (see Materials and Methods) using either RBD (Fig. 2A)-, nucleocapsid (Fig. 2B)-, or spike (S1-RBD) (Fig. 2C)-coated ELISA plates and the RBD soluble cPass sVNT (Fig. 2D). Of the PCR-positive samples (Fig 2, circles at the left side of each graph), six were categorized as false negatives and shared between three tests (nucleocapsid [Fig. 2B], spike S1 [Fig. 2C], and cPass sVNT [Fig. 2D] [red circles]). The RBD-coated ELISA (Fig. 2A) plates gave only two false-negative samples (shared between the four tests [red circles]) but also exhibited three false-positive samples (Fig. 2A, squares at the right side of graph above the cutoff line), suggesting a lower specificity for this assay. The remaining negatively classified samples (i.e., false negatives) among the 45 PCR presumed positive samples (Fig. 2, black circles at the left side of each graph below the cutoff line) for the nucleocapsid (Fig. 2B) and spike S1 (Fig. 2C) protein-coated ELISA plates were likely undetectable by these assays. For the 23 prepandemic presumed negative samples (Fig. 2, squares at the right side of each graph), the RBD (Fig. 2A)- and nucleocapsid (Fig. 2B)-coated ELISA plates misclassified 3 and 1 samples, respectively, as positive (squares above the cutoff line), whereas spike S1 (Fig. 2C) and the cPass sVNT (Fig. 2D) classified all samples correctly as negative (squares below the cutoff line). In summary, for study 1, the GenScript cPass sVNT delivered comparable or improved accuracy and negative and positive predictive values versus the other serology tests (Table 1).

For study 2, the data from previously tested human serum samples (21) (see Materials and Methods) were directly compared with the data for the cPass sVNT for SARS-CoV-2-positive (blood samples drawn at different intervals after symptom onset) and prepandemic deidentified individuals (Table 2). Consistent with study 1, the cPass sVNT gave results that were similar or superior to those of the other serological tests and also demonstrated the presence of neutralizing antibodies within 5 days after symptom onset.

A large cohort of well-characterized, PCR-verified positive and negative samples was screened with the cPass sVNT (Table 3). The comparable or superior specificity and sensitivity compared to other commercial serology tests (7, 22) translate to comparable or higher positive (94.5%) and negative (99.7%) predictive values and overall accuracy (99.2%), which is critical in population monitoring and contact tracing.

### cPass sVNT ELISA versus live-cell viral neutralization tests (PRNT and FRNT).

**FIG 3:**
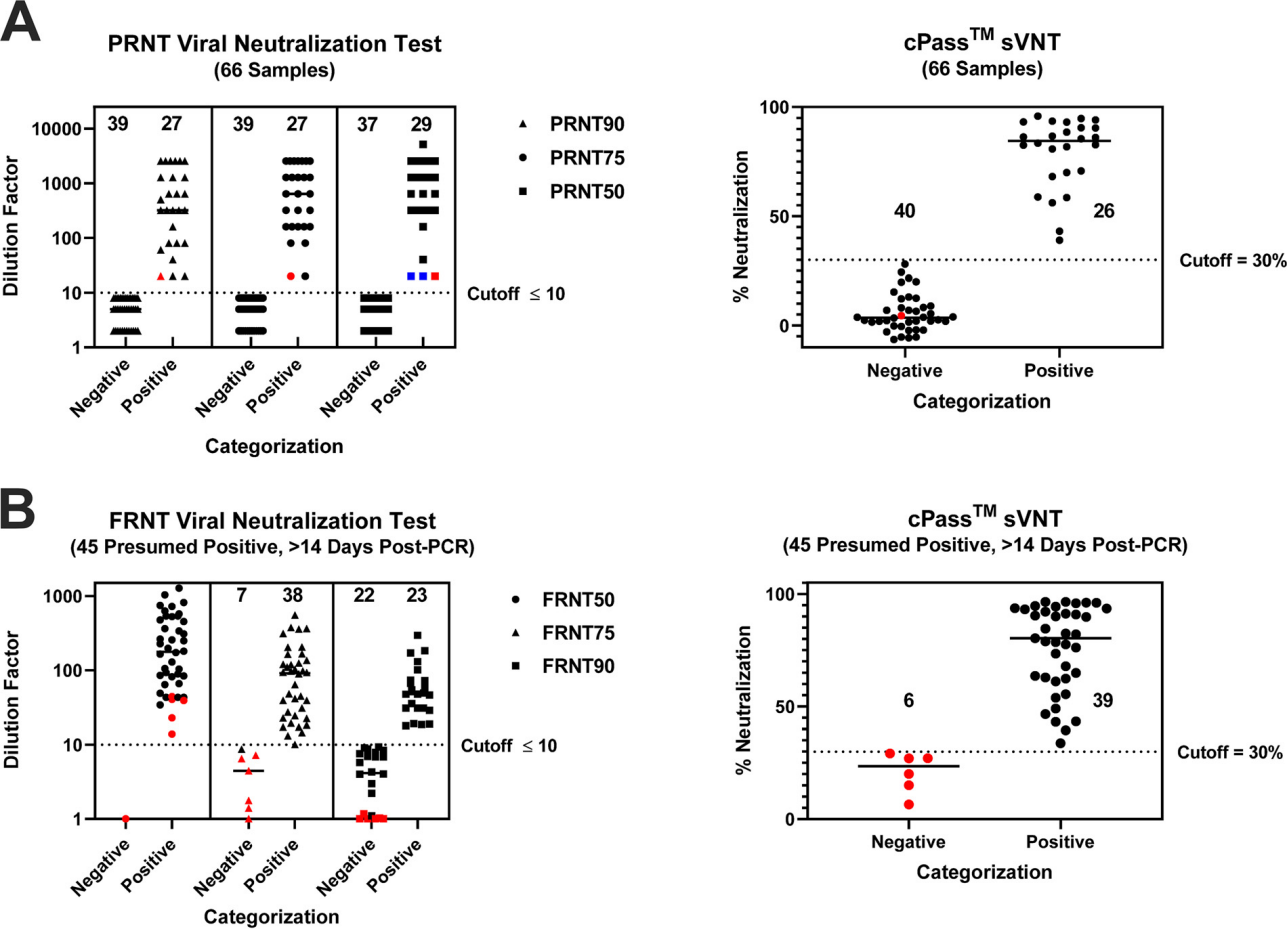
Direct comparison between the PRNT, FRNT (at different analysis stringencies), and sVNT. (A) PRNT. Sixty-six samples were assayed between the PRNT_50_, PRNT_75_, PRNT_90_, and sVNT. One sample was discordant between the sVNT and the PRNT_75_ and PRNT_90_ (red dot). Two samples were discordant between the PRNT_50_ and PRNT_75_ (blue dots). For the PRNT, negative samples with values below 10 were randomly assigned values of 2, 5, or 8 to more easily visualize the number of negative samples. (B) FRNT. Forty-five presumed positive samples were tested between the FRNT_50_, FRNT_75_, and FRNT_90_ and the sVNT. The same six samples were categorized as negative by the sVNT, FRNT_75_, and FRNT_90_ (red dots). One sample was discordant between the FRNT_75_ and sVNT. For the FRNT, all samples with a value of zero were assigned a value of 1 to more easily visualize the negative samples.

The CDC’s interim guidelines for COVID-19 antibody testing specific for SARS-CoV-2 neutralizing antibody detection include two assays for neutralizing antibody screening: (i) a virus-neutralizing test (VNT) such as the plaque reduction neutralization test (PRNT) and the focus reduction neutralization test (FRNT) and (ii) the pseudovirus neutralization test (pVNT) (23). These tests require live cells and virus with a multiday procedure that necessitates a BSL2 or BSL3 containment laboratory. Since the RBD for both SARS-CoV-1 and SARS-CoV-2 is immunodominant (8, 17), it has been postulated and shown that the cPass sVNT gives comparable results (19) and can potentially be used in lieu of the VNT or pVNT. Comparison of the PRNT_50_, PRNT_75_, and PRNT_90_ values with the sVNT values on 66 well-characterized samples gave a high correlation in delineating positive and negative samples for PRNT_75_ and PRNT_90_, where one sample (red dot) of 66 did not corroborate (Fig. 3A). However, when using a lower-stringency analysis for the PRNT (i.e., the reciprocal dilution that inhibited 50% of infection), two of the samples found to be negative by the cPass sVNT had detectable PRNT_50_ titers (Fig. 3A, blue dots).

The same 45 PCR-confirmed, presumed positive samples from study 1 (Fig. 2 and Table 1) were also tested for live-virus-neutralizing activity using a SARS-CoV-2-specific FRNT. FRNT_50_, FRNT_75_, and FRNT_90_ titers were determined, giving excellent correlation between the cPass sVNT and FRNT_75_ (Fig. 3B). However, when comparing FRNT_75_ to FRNT_50_, five of the six samples found to be negative by the cPass sVNT had detectable FRNT_50_ titers. Reciprocally, 16 samples found to be positive by the cPass sVNT did not have detectable FRNT_90_ titers.

### Temporal persistence of circulating neutralization antibodies in longitudinal studies (experimental design is critical).

**FIG 4:**
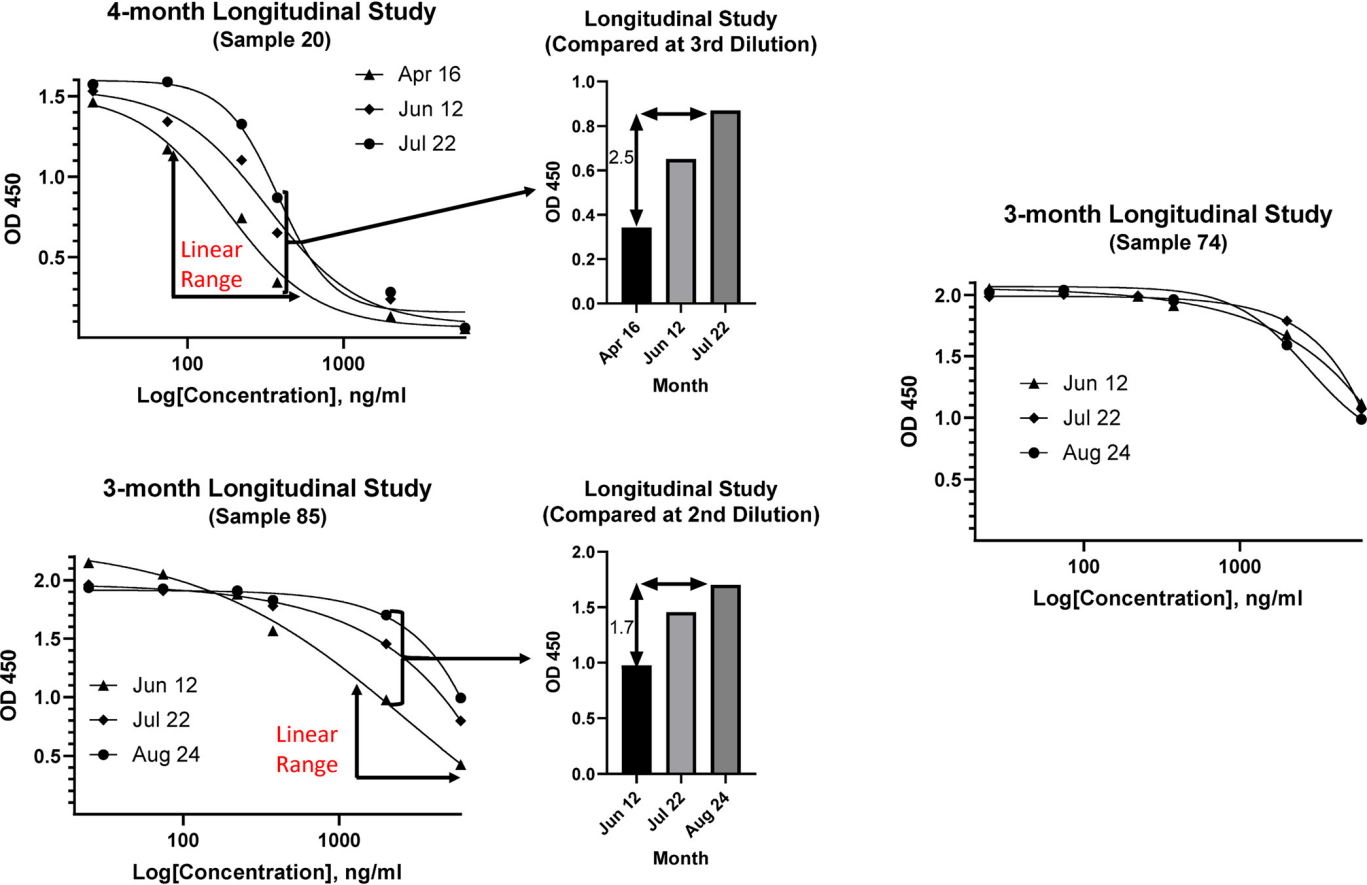
Longitudinal assessment of viral titers by the cPass sVNT for serum samples taken at different time points postinfection. Samples were initially diluted 1:10 according to the kit instructions and then serially diluted 1:3 for an additional five dilutions to generate a competition curve for each sample at each time point. Samples 20 and 85 were compared for titers within the linear range of each curve or by the OD_450_ ratio.

Serum samples from three individuals who recovered from COVID-19 were collected over 3 months to assess the persistence of inhibitory antibodies using the cPass sVNT (Fig. 4). In order to determine the true quantitative difference between the time points, a serial dilution series of each sample was performed on the same plate to uncover a dilution whereby the signals were within the linear, quantitative range. For sample 20, the third dilution (1:90) was within the linear range of each dilution series, and a decrease in inhibitory antibodies of approximately 2.5-fold was measured over a 4-month period. For sample 85, the second dilution (1:30) was within the linear range and gave a 1.7-fold decrease over 3 months. However, if the samples had been diluted by only 10-fold, where the signal was within the lower plateau of the dilution curves for all three time points, very little difference in inhibitory antibodies would be quantified for sample 20 (compare the first point at the highest concentration in the dilution series at each month for sample 20). Sample 74 exhibited almost overlapping dilution curves over 3 months, indicating no change and, thus, persistence of immunity over that time period.

## DISCUSSION

The quality of serology test data has been widely variable and resulted in lower levels of sensitivity and specificity for some commercial tests, which has led to reduced confidence in serological testing. This can directly contribute to the increased spread of disease (5, 7, 20, 22, 24). The root cause of reduced accuracy is likely a consequence of the choice of antigen, associated posttranslational modifications (25, 26), and/or the protein-coated surface of the ELISA plates. Since the process for coating ELISA plates relies primarily on hydrophobic interactions, coating plates with proteins such as the spike S1 and S2 domains, the RBD, or nucleocapsid proteins of SARS-CoV-2 can lead to various subpopulations of structurally altered antigens in each well (14). This in turn can lead to the exposure of antigenic sites that would not otherwise be present in the native state, giving increased false positives from nonspecific immunoglobulin binding (11, 15). This was observed for nucleocapsid- and RBD-coated plates with prepandemic samples in study 1 (Fig. 2A and Table 1) and for the Abbott and DiaSorin tests in study 2 (Table 2). This issue has also been described for other serology assays (4, 7, 22, 24). Ideally, the “bait” protein used to capture circulating immunoglobulins should be in a native or near-native conformation to ensure that the antigenic sites of the protein are correctly and consistently exposed to the disease-related antibodies. This is likely the case for the cPass sVNT because the purified RBD-HRP is supplied and applied in solution (Fig. 1) (16) and evidenced by the high specificity of the assay in this work (Tables 1 to 3). Although the cPass sVNT utilizes hACE2 protein-coated plates, which can lead to structural perturbations of this protein, there is evidence that the immobilized hACE2 receptor maintains a strong interaction with the RBD, suggesting minimal loss of structural integrity (16, 26–28). Furthermore, since immunoglobulins from any isotypes that recognize RBD antigenic sites can bind and will be measured as a total antibody response, the sensitivity and negative predictive value of this versus immunoglobulin-specific tests (i.e., IgG/IgM) should be similar or improved for the cPass sVNT, as was shown here (Fig. 2 and Tables 1 and 2) and by others (29).

Taken together, these points help explain the similar or improved specificity, sensitivity, positive and negative predictive values, and accuracy obtained for the cPass sVNT versus other popular commercial SARS-CoV-2 IgG tests (Tables 1 and 2) (7, 29). Furthermore, these data support the notion that a binding antibody response as measured by the presence of circulating immunoglobulins coincides with neutralizing antibodies.

Although for study 1, the cPass sVNT categorized a total of six PCR-positive samples as negative (Fig. 2D, red circles), the spike (S1) (Fig. 2C) and nucleocapsid (Fig. 2B) assays also coincided with their negative classification, suggesting that these “false negatives” were, in fact, true negatives or possibly did not seroconvert. Since these samples were categorized as positive by quantitative PCR (qPCR) testing, this raises the question about the accuracy of qPCR. Some recent SARS-CoV-2 and Middle East respiratory syndrome (MERS) studies suggest that rates of PCR false positives can range from about 2% to 30%, with an average of 8% (30, 31). This may be attributed to using a cycle threshold cutoff that is too high and beyond the limit of detection for qPCR (6), accounting in part for the six false-negative samples delineated by the cPass sVNT and the other two serology tests (Fig. 2B to D, red dots). In fact, at quantification cycle (*C_q_*) values above 35, many of the technical replicates for a given sample are negative, and single copies of contaminating DNA can result in a false-positive call (32).

### Application of the cPass sVNT as a high-throughput screening tool for COVID-19 drug or vaccine development.

In order to abrogate viral entry, replication, and spread of infection, a vaccine should induce the production of antibodies that block (or neutralize) the interaction between the RBD and the hACE2 receptor (33–35). Some antibody-based drug candidates are similarly targeting this interaction (36, 37).

To date, the gold and silver standards in assessing the neutralization activity from drugs or antibodies are viral neutralization tests (VNTs) and pseudovirus neutralization tests (pVNTs) (38, 39). The VNT requires live SARS-CoV-2 and cells that express the hACE2 receptor and therefore requires a BSL3 containment laboratory, personal protective equipment, and highly trained personnel to conduct the experiments, whereas the pVNT can be performed in a BSL2 laboratory. Both tests involve sample incubations and manipulations that give results in 2 and 4 days and are therefore of relatively low throughput, expensive, and time-consuming, requiring aseptic techniques and personal protective equipment. The early phases of vaccine or drug development typically require the screening of large numbers of compounds and/or serum samples from candidate vaccine clinical trials to uncover those that neutralize the virus-host cell interaction with the greatest efficiency and efficacy (40). Furthermore, once a good potential vaccine or drug candidate has been selected, clinical trials involving thousands of individuals are required to assess protection against infection and the longevity of the neutralizing antibody response postvaccination (41). Thus, thousands of samples must be collected at regular time points and screened for neutralizing antibody titers, which would be challenging, expensive, and time-consuming using the VNT or pVNT.

Since the cPass sVNT utilizes the purified protein components of the RBD-hACE2 interaction in a high-throughput ELISA requiring about 1.5 h for each 96-well plate assay in a BSL2 laboratory (Fig. 1), it can potentially be used to screen for the best neutralizing drug and/or antibodies generated by vaccination (16). The cPass sVNT was compared directly with the FRNT and PRNT using serum from patients who recovered from COVID-19. An excellent correlation with FRNT_75_, PRNT_75_, and PRNT_90_ in detecting the presence of neutralizing antibodies postinfection (Fig. 3) (19) was revealed, supporting its application as a reliable tool for vaccine development and longitudinal studies tracking immune responses postvaccination. These data are likely owing to the immunodominance of the RBD versus other antigenic sites of the spike protein (8, 9).

There is no consistency in the literature concerning the analysis stringency that should be applied to cell-based neutralization assays (i.e., PRNT_50_ versus PRNT_90_ or FRNT_50_ versus FRNT_90_) to ensure the accurate delineation of positive and negative samples. Recent concerns have emerged concerning the ensuing variability and confidence in the results when different stringencies are applied to the data analysis of these live-cell assays (42, 43). The correlation of the cPass sVNT with the FRNT and PRNT was examined between 50% and 90% foci and plaque reduction. Significant changes in the analysis were observed between the PRNT_50_ and PRNT_75_ (Fig. 3A shows two samples shifting from negative to positive [blue dots]) but with no change between the PRNT_75_ and PRNT_90_. For the FRNT, there were large differences between the FRNT_50_, FRNT_75_, and FRNT_90_ (Fig. 3B), making it difficult to accurately determine the true delineation of positive and negative samples. The corroboration of the cPass sVNT with the higher-stringency PRNT_75_ and PRNT_90_ (Fig. 3A) is supported by recent work (19) and underlines the benefit of this test in accurately delineating neutralization antibody-positive and -negative individuals.

### Experimental design for comparative drug or vaccine testing.

The cPass sVNT was used to assess dynamic changes in neutralizing antibodies from samples from recovered SARS-CoV-2 patients. Within the linear range, there was a significant decrease in inhibitory antibodies over time for samples 20 and 85, with no decrease observed for sample 74 (Fig. 4). However, outside the linear range, near the upper or lower plateau of the dilution curves, the data points for each time point were almost overlapping. This underlines the importance of producing a dilution series from each sample when dissecting the quantitative difference in neutralization titers over time. The cPass sVNT offers a much-higher-throughput, lower-cost, and safer option to achieve high-quality longitudinal data versus the more traditional VNT and pVNT, especially considering the close correlation with the high-stringency PRNT_90_ (19).

### Conclusion.

The cPass sVNT provides a newly structured, high-throughput assay (Fig. 1) that permits augmented specificity, sensitivity, and accuracy for serological assessment of disease versus preexisting IgG tests (Fig. 2 and Tables 1 to 3) (7). The test also permits the functional delineation of virus neutralization for patient recovery that correlates strongly with live-cell neutralization (PRNT_90_) (Fig. 3) (19) and for high-throughput screening of drug and vaccine immune response antibodies that neutralize the interaction between the RBD and the hACE2 receptor (Fig. 4).

### Regulatory status.

The cPass SARS-CoV-2 neutralization antibody test is CE marked for diagnostic use in the European Union and authorized for emergency use by Health Sciences Authority in Singapore and the U.S. Food and Drug Administration for qualitative delineation between positive and negative patient samples. The quantitation and automation protocols have not yet been authorized by the FDA, the European Union, or Singapore and are for research use only.
